# Microdroplet-based On-Demand Drawing of High Aspect-Ratio Elastomeric Micropillar and Its Contact Sensing Application

**DOI:** 10.1038/s41598-017-17230-3

**Published:** 2017-12-05

**Authors:** Qiang Li, Rabin Dhakal, Jaeyoun Kim

**Affiliations:** Department of Electrical and Computer Engineering, Iowa State University, Ames, IA 50011 USA

## Abstract

High aspect-ratio elastomeric micropillars play important roles as the platform for microscale sensing and actuation. Many soft-lithographic techniques have been developed for their facile realization but most of the techniques are limited to build the micropillars only on totally flat, widely accessible substrate areas with the micropillar’s structural characteristics completely predetermined, leaving little room for *in situ* control. Here we demonstrate a new technique which overcomes these limitations by directly drawing micropillars from pipette-dispensed PDMS microdroplets using vacuum-chucked microspheres. The combined utilization of PDMS microdroplets and microspheres not only enables the realization of microsphere-tipped PDMS micropillars on non-flat, highly space-constrained substrate areas at *in situ* controllable heights but also allows arraying of micropillars with dissimilar heights at a close proximity. To validate the new technique’s utility and versatility, we realize PDMS micropillars on various unconventional substrate areas in various configurations. We also convert one of them, the optical fiber/micropillar hybrid, into a soft optical contact sensor. Both the fabrication technique and the resulting sensing scheme will be useful for future biomedical microsystems.

## Introduction

High aspect-ratio (HAR) micropillars have been attracting an increasing level of research interest thanks to their unique mechanical and optical properties. They are already playing crucial roles in biomimetic dry adhesives^1,2^, super-hydrophobic surfaces^3–5^, microneedles for drug delivery^6^, tunable optical windows^7,8^, actuators^9^, air flow sensors^10^, and nanonewton-scale force sensors^11,12^. A number of materials have been utilized for the realization of HAR micropillars^13^. Of special interest among them is poly(dimethylsiloxane) (PDMS) which functions as the primary building material for microfluidic, soft-robotic, and biomedical devices owing to its compatibility with soft-lithographic rapid prototyping, transparency in the visible and infrared spectral regimes^14^, high-level deformability^15^, gas-permeability, and bio-compatibility^16,17^.

Several methods have been developed for PDMS HAR micropillar fabrication. The most common technique is replica-molding in which liquid-phase PDMS is poured over a mold bored with HAR holes and then cured to form micropillars^18^. In this technique, however, the completed HAR micropillars must be physically peeled off from the mold. Since the risk of structural failure increases with the height and thinness of the PDMS micropillar, the ratio between the micropillar’s height and average diameter achievable with replica-molding has been limited to ~20^12^. In addition, the inherently low Young’s modulus of PDMS^19^ further complicates the fabrication by causing the HAR micropillars to collapse irreversibly to the substrate during and after the demolding process^20,21^.

Recently, we demonstrated a new technique which overcomes the two challenges by directly drawing the micropillars from a spin-coated PDMS thin film while applying thermal curing and hardening to the micropillars *in situ*. Using the technique, we realized PDMS micropillars with their aspect-ratios exceeding 100^10^. The technique was also unique in utilizing microspheres as the drawing probe. Since the microsphere drawing probe is self-aligned to the micropillar and can be left on the tip of the micropillar after the drawing, it can add various functionalities to the completed PDMS HAR micropillar^10^. For instance, in our previous work, we adopted a silver-coated microsphere as the drawing probe so that it can later function as a self-aligned mirror to the lightwave guided by the transparent PDMS micropillar lightguide.

However, most of the PDMS micropillar fabrication techniques, including our direct drawing-based one, suffer from three important limitations. First, they all require wide, highly accessible space on a totally flat substrate to accommodate complex soft-lithographic processes such as spin-coating of PDMS thin films and/or physical peel-off of the cured PDMS layer. To widen the application scope of the HAR micropillars, the fabrication technique must be applicable to surfaces that are non-flat and/or with strong constraints on space and accessibility. Second, in most techniques, the micropillar’s structural characteristics, such as the height and thickness, are predetermined at the stage of mold preparation, leaving little room for *in situ* control. Third, even in the few techniques which allow *in situ* control of some of the micropillar’s structural characteristics, such as our direct drawing-based technique which allows *in situ* control of the micropillar’s height, the control applies to the entire micropillar array under fabrication, prohibiting neighboring micropillars from exhibiting dissimilar characteristics.

Here we present a new, direct drawing-based HAR micropillar fabrication technique which addresses all three issues stated above. It builds PDMS micropillars with *in situ* controllable heights on non-flat, highly space-constrained areas. It also allows arraying of micropillars with dissimilar heights at a close proximity to each other.

There are three key enabling factors. First, as the source of the PDMS for the direct drawing, we adopt pipette-dispensed PDMS microdroplets, rather than the spin-coated PDMS thin films used in our previous work^10^. This eliminates the need for spin-coating which inevitably necessitates a widely open area on a flat substrate. In addition, it also helps isolating micropillars from their neighboring ones. In the case of PDMS thin film-based micropillar drawing, all the micropillars in one batch were inevitably coupled at their bases and the resulting interaction between the neighboring micropillars rendered their tight packing very difficult. The use of isolated PDMS microdroplets fundamentally eliminates these limitations. Second, to grab and hold the microsphere during the micropillar drawing, we choose to employ a pipette-based vacuum-chuck^22^. Compared with the double-stick tape we used in our previous work^10^, it makes the release of the microsphere much more controllable and faster. It also greatly facilitates the visually guided micropillar positioning process by obstructing much narrower field of view than the double-stick tape does. Third, to facilitate and expedite the *in situ* thermal curing and hardening of the PDMS micropillars, we change the source of the heat from the hot plate^10^ to ultraviolet (UV) illumination which is more controllable and efficient owing to the high absorption coefficient of PDMS at the UV regime^23^.

To validate the versatility of our new technique, we fabricate HAR PDMS micropillars on the end-facets of glass optical fibers and on the side of a 1 mm-diameter cylindrical polymer fiber. Furthermore, we array micropillars with different heights with narrow pillar-to-pillar spacing, which will be useful for multi-level flow sensing^24^. As an exemplary application, we also configure the optical fiber/micropillar hybrid structure into a soft optics-based contact sensor with flexible elastomeric waveguide and self-aligned reflector^25^.

## Methods

### Preparation of PDMS microdroplets

**Figure 1:**
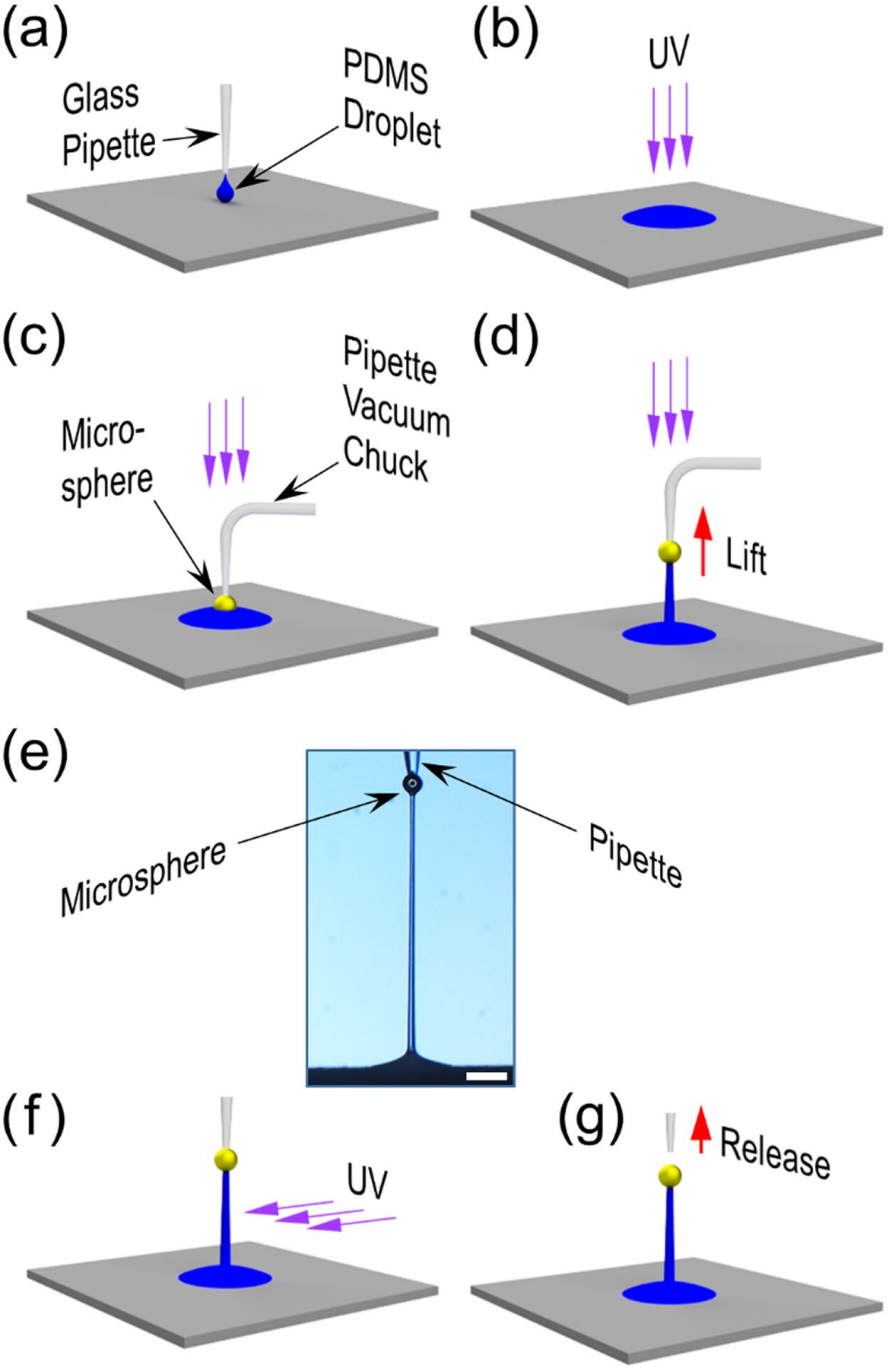
Microdroplet-based PDMS micropillar direct drawing steps. (**a**) A liquid-phase PDMS microdroplet is dispensed onto the target area through a position-controlled pipette. (**b**) Partial UV curing of the PDMS microdroplet. (**c**) A pipette-chucked microsphere is lowered until it makes a physical contact with the PDMS microdroplet. (**d**) The microsphere is lifted to the desired height. The UV curing light may or may not be illuminated. (**e**) The optical micrograph of a PDMS micropillar drawn by the pipette-chucked microsphere. (Scale bar: 100 µm). (**f**) Post-curing of the PDMS micropillar with side-illuminated UV light. (**g**) Release of the micropillar through termination of the vacuum pump operation.

The PDMS pre-polymer base and its crosslinking curing agent (Sylgard 184, Dow Corning) were mixed at 10:1 weight ratio, then degassed for 40 mins for air bubble removal. Increasing the weight ratio of the curing agent may lead to stiffer micropillars after the drawing^19,26^. The study to relate the PDMS composition and the mechanical characteristics of the final micropillar structure is left as a future work. To generate the PDMS microdroplets, we first let the liquid-phase PDMS fill a 20 µm orifice glass pipette (MGM 1C-20, FivePhoton Biochemicals) via capillary action. Then we dispensed the liquid-phase PDMS from the pipette onto the target area using pulsed pressure generated by a time-controlled precision pump (PV830 Pneumatic PicoPump, World Precision Instrument), as shown in Fig. 1a. The pulse duration and frequency were approximately 20 ms and 3 Hz, respectively. The dispensed PDMS formed microdroplets with approximately 25 μm and 500 μm in central height and diameter, respectively. Then the PDMS microdroplet was partially cured for 70 s under ultraviolet (UV) illumination generated by a commercial curing system (BlueWave 200, Dymax), as depicted in Fig. 1b. The center wavelength of the light was 366 nm (UVA) and the light intensity was set to approximately 1.0 W cm^−2^. Since the glass pipette was mounted on a precision micromanipulator (MP-285 ROE, Sutter Instrument), we could dispense the PDMS microdroplets with spacing as narrow as 500 μm.

### Vacuum-chucking of microspheres

As the drawing probes, we chose 25~60 μm-diameter Ag-coated hollow glass microspheres from Cospheric (M-40, 0.49 g/cc). As the vacuum-chuck for the microspheres, we utilized right-angled glass pipettes (FivePhoton Biochemicals) so that we could illuminate the UV light on the PDMS micropillar from the top during the direct drawing and achieve a more uniform curing (Fig. 1c). For accurate alignment of the microsphere with respect to the PDMS microdroplet, we controlled the position of the glass pipette with the micromanipulator (MP-285 ROE, Sutter Instrument). Two microscopes were used to monitor the alignment process from two mutually orthogonal directions.

### Direct drawing of PDMS micropillar

Upon completing the partial curing of the PDMS microdroplet, we lowered the pipette-chucked microsphere until it made a contact with the PDMS microdroplet (Fig. 1c). After maintaining the contact for approximately 120 s, we lifted the microsphere to the desired height that can be controlled *in situ* (Fig. 1d). The contact time was set by taking two factors into consideration. On the one hand, the contact time should be long enough to ensure good adhesion between the microsphere and the PDMS microdroplet. Otherwise, the microsphere might be detached from the PDMS during the subsequent drawing process. On the other hand, the contact time should be short enough to prevent excessive solidification of the PDMS microdroplet which will encapsulate the microsphere in the PDMS, also disabling the subsequent drawing process. The pre-programmed lift-up speed was typically 100–200 μm/s. Figure 1e shows the optical micrograph of a PDMS micropillar after the lifting-up step. The PDMS micropillar was then post-cured for approximately 4 hours by the side-illuminated UV light as shown in Fig. 1f. For the post-curing, the UV light intensity was set to ~2.0 Wcm^−2^. Once the micropillar became completely cured, the microsphere was released by terminating the vacuum-chuck operation as shown in Fig. 1g. In our previous work, the microsphere-tipped micropillars had to be maintained at 200% stretching until the microspheres got detached from the double-stick tape, which could take hours^10^. This pipette-based vacuum-chucking of the microsphere has rendered the release an instant, reliable, and highly controllable process.

## Results

### Drawing and arraying of dissimilar micropillars

**Figure 2:**
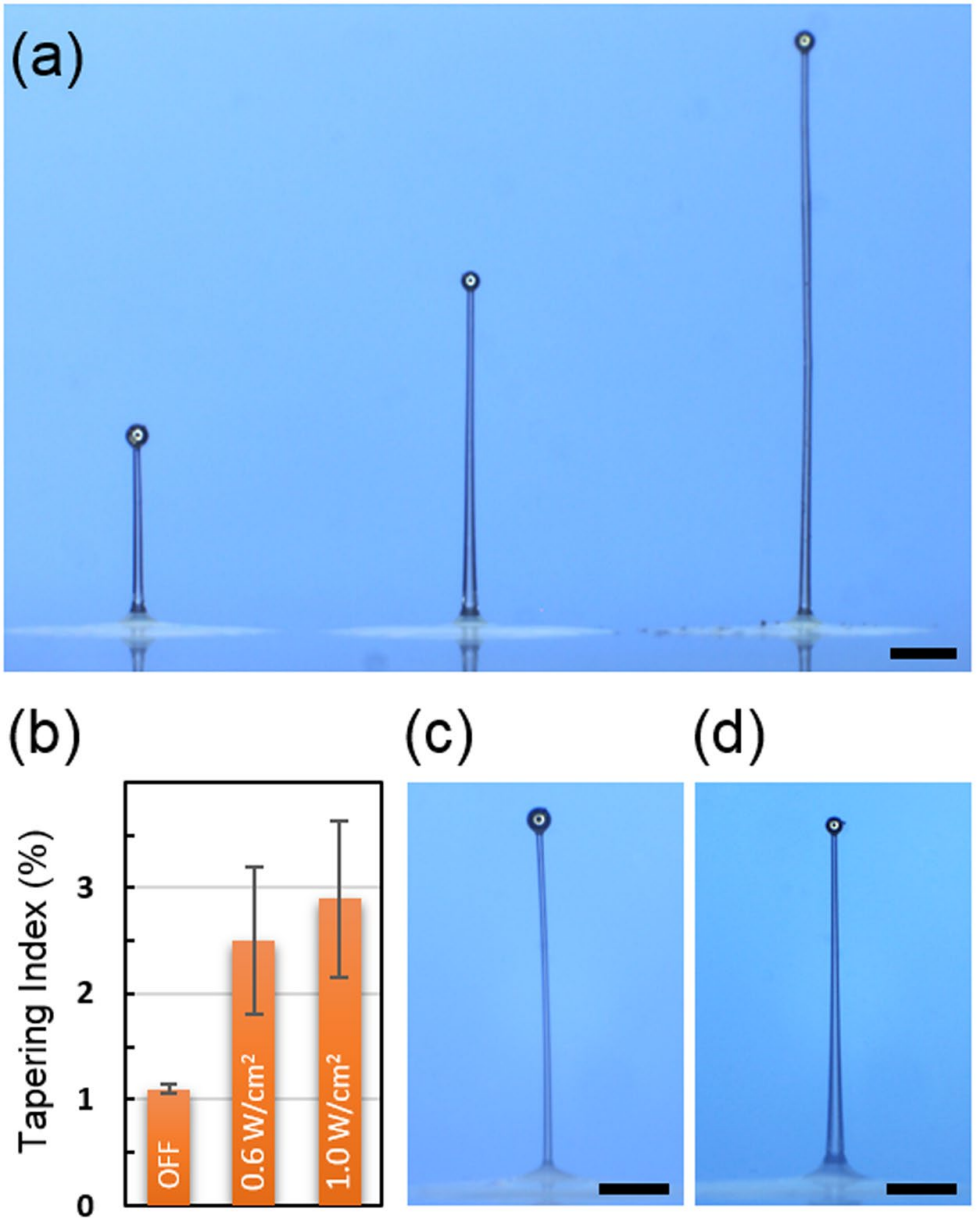
Arraying and shape-control of microsphere-tipped PDMS micropillars. (**a**) Three PDMS micropillars closely packed while exhibiting highly different structural characteristics. The heights are (from left to right) 454, 900 and 1570 µm, respectively. With the corresponding average diameters at 23.5, 27.0, and 28.4 μm, the aspect ratios are 19.3, 32.6 and 54.6. The micropillars are separated by ~900 µm. (Scale bar: 200 µm). (**b**) The tapering indices (TI) of PDMS micropillars drawn with three different levels of UV curing light intensity during the drawing process (Fig. 1d). The error bars represent the standard deviation from 3~5 samples. (**c**) The optical micrograph of a PDMS micropillar with the *in situ* UV curing light OFF. The tapering index is 1.0%. (Scale bar: 100 μm). (**d**) The optical micrograph of a PDMS micropillar drawn with the *in situ* UV curing light ON. The tapering index is 3.7%. (Scale bar: 100 μm).

To demonstrate the versatility of our new fabrication technique, we fabricated multiple micropillars with dissimilar structural characteristics at close proximity. As shown in Fig. 2a, three micropillars were directly drawn in a row from three pre-dispensed PDMS microdroplets. Each microdroplet was approximately 25 μm and 500 μm in central height and diameter, respectively, and separated from its direct neighbor by 900 μm. The heights of the PDMS micropillars were set by controlling the micromanipulator’s programmable vertical displacement during the drawing process. For the present work, we set the vertical displacement to 460, 900, and 1570 μm and obtained 454, 881, and 1550 μm in the micropillar’s height, respectively. With the average diameters at 23.5, 27.0, and 28.4 μm, the corresponding aspect ratios were 19.3, 32.6, and 54.6, respectively. We attribute the shorter heights than the preset vertical displacements to the shrinkage of PDMS during post-curing^27^. This capability to fabricate micropillars with different heights in an arrayed format has been highly sought after for various applications including multilevel near-wall shear-stress sensing^24^. The minimum separation between the PDMS micropillars is primarily determined by the minimum achievable size of the PDMS microdroplet since microdroplets within near-contact distance are highly likely to merge and negate the effort to draw the micropillars independently. The microdroplet size, in turn, depends critically on the precision of the air pressure pulse duration and the minimum PDMS volume required to realize the targeted micropillar height. Even though these micropillars were drawn by a new technique, their material composition, shape, and dimensions are very similar to those realized in our previous work and very likely to exhibit similar optomechanical characteristics quantified in the report^10^.

### UV-assisted fine-tuning of micropillar shape

Along the fabrication trials, we discovered that the presence of the UV curing light during the micropillar drawing, shown in Fig. 1d, strongly affects the overall shape of the completed micropillar. In particular, we found that the level of tapering along the micropillar is severely impacted. For quantitative analysis, we define the tapering index (*TI*) as

1 $$TI\equiv ({d}_{base}-{d}_{top})/h\times 100[ \% ]$$

where *d*_*base*_, *d*_*top*_, and *h* are the base diameter, the top diameter, and the micropillar height, respectively, and collected data from multiple samples drawn under three different levels of *in situ* UV curing light. The calculated *TI* value reached average values of 1.1%, 2.5%, and 3.1% for 0.0, 0.6, and 1.0 W·cm^−1^, respectively, as shown in Fig. 2b. The corresponding standard deviations were 0.04% (*n* = 3), 0.70% (*n* = 5), and 0.74% (*n* = 4), respectively. The representative optical micrographs of two micropillars with different *TI* values are shown in Fig. 2c,d. We attribute the higher *TI* value to the additional hardening of PDMS induced by the *in situ* UV curing which, in turn, leads to a larger base diameter.

Repeated experiments revealed, and the results from our previous work corroborate, that the final structural characteristics, such as the aspect ratio, are determined mainly by the required UV dose rather than the drawing rate, or equivalently, the drawing time. The relation between the microsphere’s structural characteristics and the liquid’s rheological properties on the subsequent formation of the *capillary bridge*, which eventually becomes the micropillar through curing and hardening, is a topic of intense research with many theories and models presented for further studies^28–31^.

### Micropillar drawing on unconventional substrates

**Figure 3:**
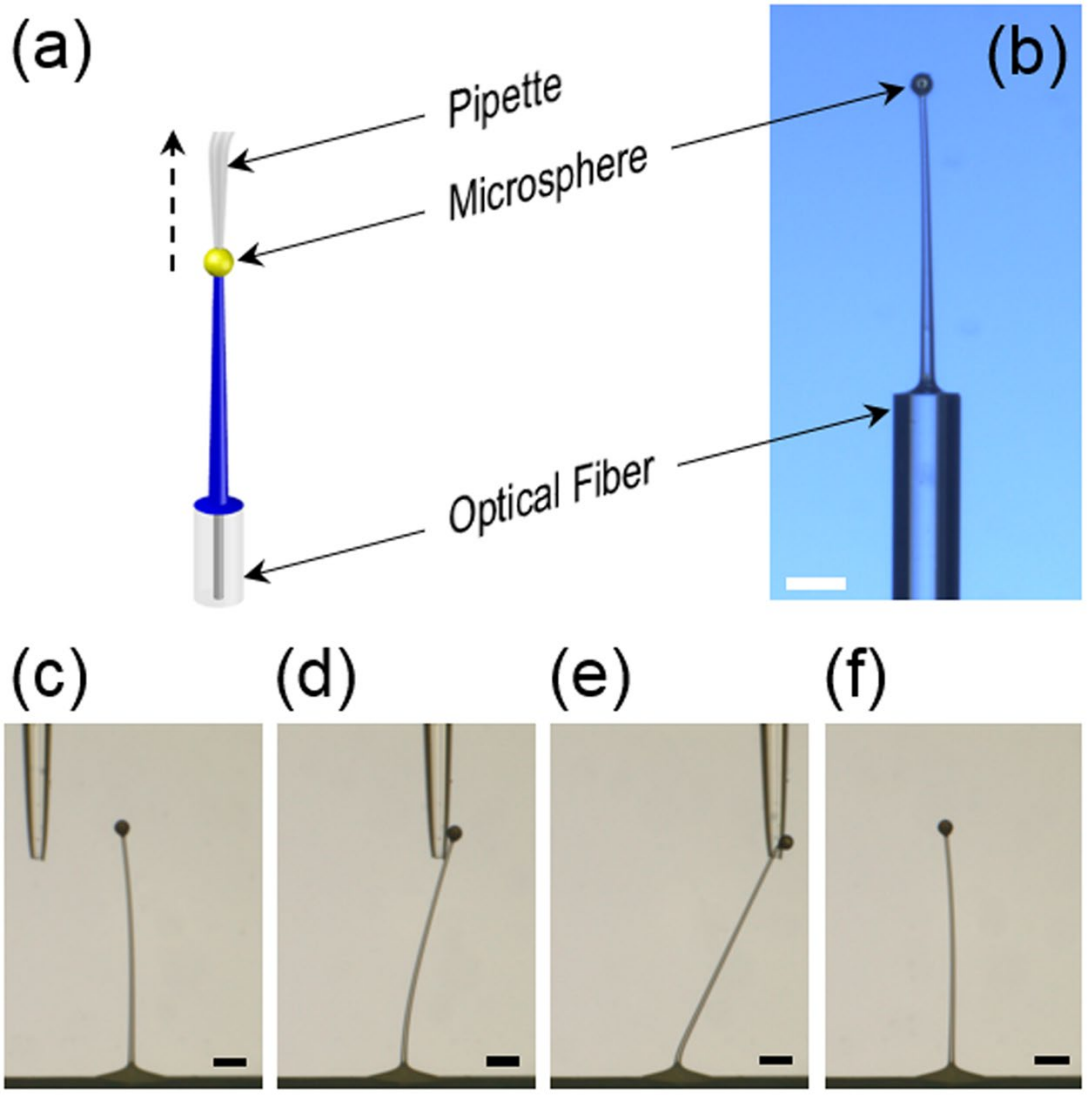
Direct drawing of PDMS micropillars on space-limited substrates. (**a**) A schematic diagram of the PDMS micropillar drawing from the end facet of a glass optical fiber. (**b**) The optical micrograph of a 484 µm-high PDMS micropillar drawn on end facet of a 125 µm-diameter glass optical fiber. Its aspect ratio is 28.5. (Scale bar: 100 µm). (**c**–**f**) The nudging experiment to test the completed micropillar’s robustness and its adhesion to the substrate. A glass pipette was utilized to tilt the micropillar on PTFE surface. (Scale bars: 100 μm).

Drawing of PDMS micropillars on non-flat, highly space-limited areas was also set as a goal in this work. To demonstrate its accomplishment, we fabricated PDMS micropillars on two different unconventional substrates. The first was the end facet of the standard glass optical fiber (SMF-28–100, Corning) with 125 μm outer diameter. The process is shown schematically in Fig. 3a. As depicted in Fig. 3b, the completed PDMS micropillar was 484 μm-high with its aspect-ratio at 28.5. Using two microscopes monitoring from mutually orthogonal directions, we positioned both the PDMS microdroplet and the microsphere drawing probe as close to the center of the optical fiber’s core as possible so that the resulting PDMS micropillar became coaxial with the optical fiber. Such a coaxial alignment between the PDMS micropillar and the optical fiber core will play a crucial role in utilizing the optical fiber/micropillar hybrid structure as an optical contact sensor in the following subsection.

The excellent adhesion of the PDMS pillar to glass substrates has already been confirmed in our previous report^10^. So was the adhesion between the PDMS micropillar and the metal (Ag)-coated microsphere^10^. Through this work, we found that the PDMS micropillars exhibit good adhesion to the more slippery PTFE surface as well. The completed PDMS micropillar not only withstood all the drawing and subsequent procedures but also displayed strong resilience against external forces, as can be seen in Fig. 3c–f. There, a micropipette nudges and tilts a PDMS micropillar drawn on PTFE surface but the micropillar always returns to its original position and shape upon release of the force.

**Figure 4:**
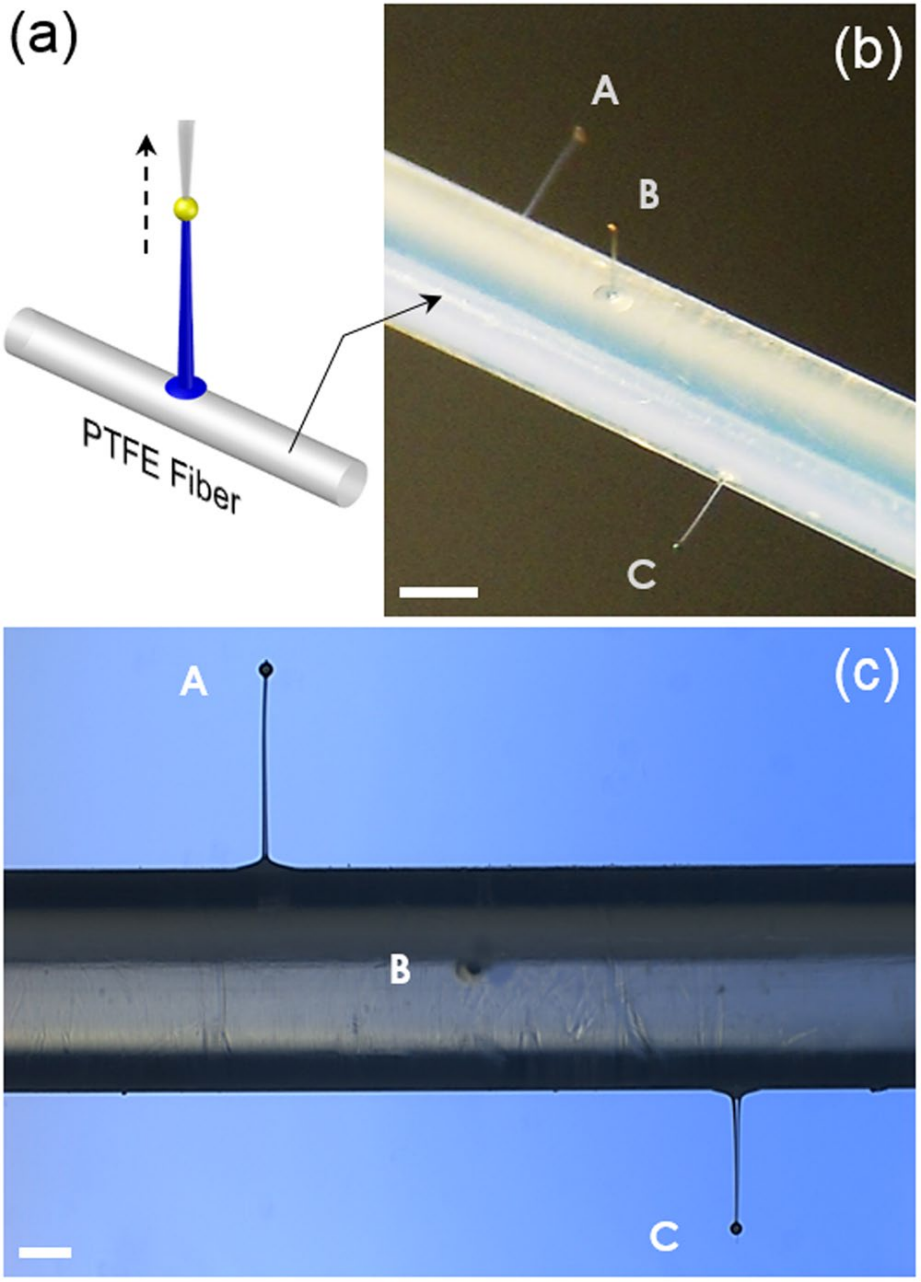
Direct drawing of PDMS micropillars on curved substrates. (**a**) A schematic diagram of the PDMS micropillar drawing from the curved side area of a thin polymer fiber (Drawn not to scale). (**b**) The bird’s eye-view optical micrograph of three PDMS micropillars drawn on the side of a 1 mm-diameter PTFE fiber. The heights and aspect ratios of A, B, and C are 726 µm, 617 µm, and 510 µm and 35.0, 28.5, and 24.8, respectively. Along the axial direction, they are separated by ~0.8 mm (Scale bar: 500 µm). (**c**) The side-view of the micropillar array in (**b**) (Scale bar: 200 µm).

The second unconventional substrate we targeted was the curved side area of a thin polymer fiber. The process is shown schematically in Fig. 4a. Specifically, we chose a 1 mm-diameter PTFE fiber and tried to draw PDMS micropillars on its broad side. In the process, we tried to diversify not only their heights but also their orientations. The latter was achieved by revolving the PTFE fiber along its axis before drawing a new micropillar. Figure 4b shows three PDMS micropillars fabricated with 726 µm, 617 µm, and 510 µm in height and 35.0, 28.5, and 24.8 in aspect-ratio, respectively. The orientation of each micropillar was successively varied by ~90 degrees. The optical micrographs of the 726 μm- and 510 μm-high micropillars are shown in Fig. 4c. The feasibility of this “curved surface drawing” approach is mainly set by the minimum size of the attainable PDMS microdroplet. The substrate fiber’s diameter must be greater than that and the fiber surface’s radius of curvature must be large enough to guarantee stable formation and sustenance of the PDMS microdroplet.

### Soft optics-based contact sensing

Many biomedical procedures require insertion of externally controlled devices into the human body. To avoid damaging the biological tissues during the insertion and the subsequent manipulation processes, it is imperative to obtain tactile feedbacks from the devices, especially from their terminal points^32–34^. Since such contact sensing itself must be performed in a safe and non-intrusive fashion, its realization poses a significant technical challenge. Optical contact sensors made of soft materials are ideal for such applications. The compliance of the material greatly reduces the risk of tissue damage. Adoption of optical sensing eliminates the concerns over electromagnetic interference, spark-generation, and electric shock. The realization of soft optics-based contact sensing, however, requires integration of rigid optical elements, such as the optical fiber, with soft optical transducers, such as the micropillar, in an accurately aligned, yet robust, fashion.

**Figure 5:**
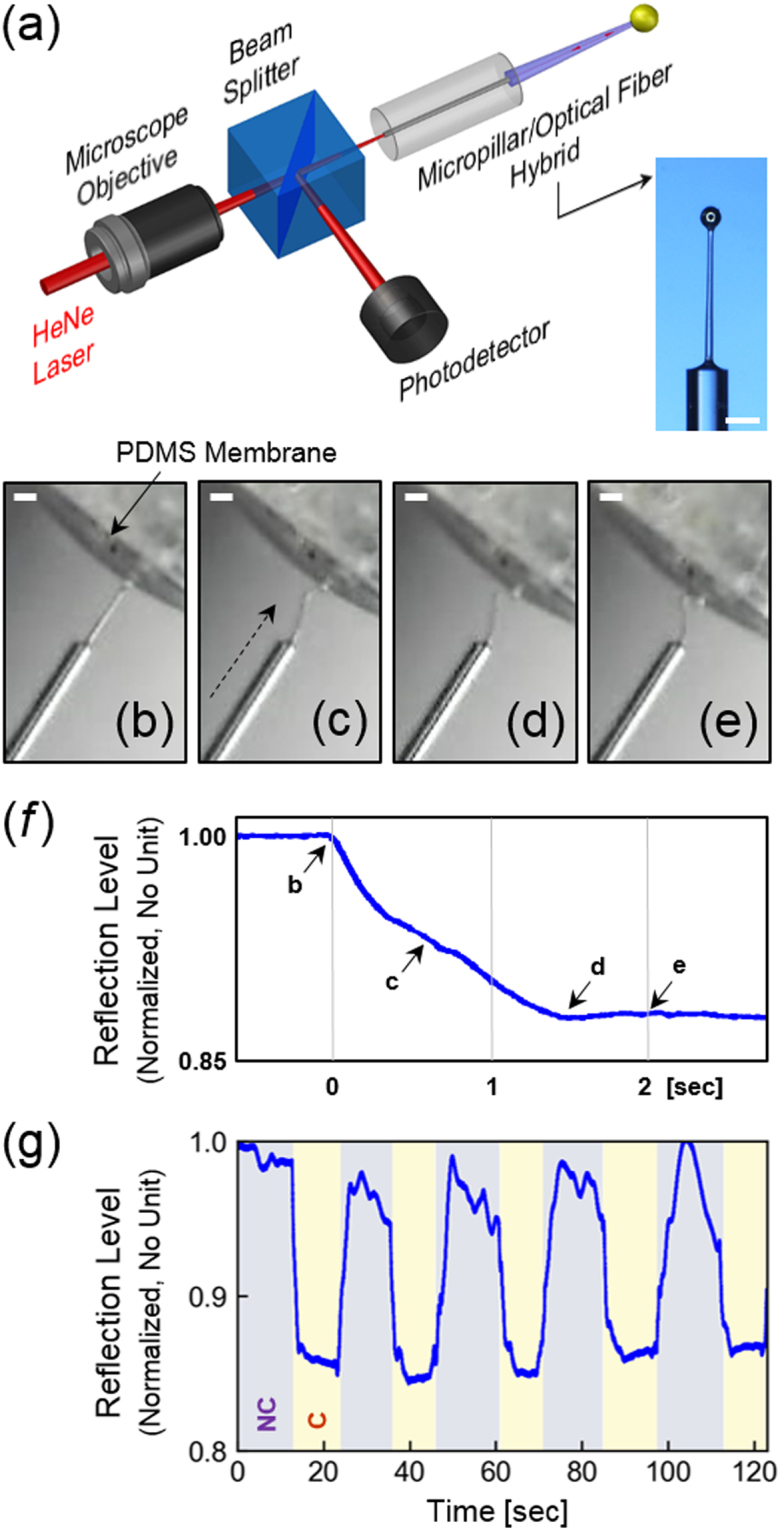
Application as a soft optical contact sensor. (**a**) Basic configuration of the contact sensor based on the optical fiber/PDMS micropillar hybrid of Fig. 3a. When straight, the PDMS micropillar maximally reflects the probe light. When in contact with an object, the flexible PDMS micropillar buckles, resulting in a drop of the reflection level. Inset: The optical micrograph of the optical fiber/PDMS micropillar hybrid used for this application. It is 412 µm high and 18.4 µm thick, exhibiting an aspect ratio of 22.4 on the end facet of a 125 μm-diameter optical fiber. (Scale bar: 100 μm). (**b**–**e**) Microscope images of the PDMS micropillar getting deformed as it was gradually pushed towards the PDMS membrane. (Scale bars: 125 μm). (**f**) The change in the reflection level as a function of the deformation level corresponding to **b**–**e**, (**g**) The change in the reflection level recorded over 5 cycles of contact/release. The dips correspond to the “contact” (C) state which deformed the PDMS micropillar maximally. NC stands for the “Non-Contact” state.

Here, as another validation of the *in situ* controlled, pipette-based PDMS micropillar drawing technique’s utility, we configured the HAR PDMS micropillar/optical fiber hybrid structure, demonstrated in Fig. 3b, into a soft optics-based contact sensor. The overall scheme is shown in Fig. 5a. The probe light from a HeNe laser (632.8 nm, 0.95 mW, Thorlabs) is first coupled into a section of multimode optical fiber (50-µm core diameter, FG050LGA, NA 0.22, Thorlabs) through a microscope objective lens (10×, NA 0.25, Newport) and a beam splitter. The alignment between the optical fiber’s core and the PDMS micropillar stated in the previous subsection greatly facilitates the coupling of the light from the optical fiber into the micropillar which functions as a flexible waveguide. Our previous results showed that 80∼120 μm radius PDMS wires can withstand >50 mN in pulling force and >400% elongation while exhibiting <3 dB/cm optical loss at 632.8 nm in wavelength^15^. The current PDMS micropillars are likely to exhibit similar characteristics.

Since the microsphere at the tip is metal-coated and self-aligned to the PDMS micropillar, it can effectively reflect the light guided by the PDMS micropillar waveguide. The reflection was then redirected by the beam splitter into the photodetector (918D-SL, Newport). We chose to use multimode optical fiber so that the light reflected from the microsphere can be easily coupled back into the optical fiber and detected. The highly flexible HAR PDMS micropillar bends when it contacts an obstructing structure. Owing to the extreme compliance of the HAR PDMS micropillar, even a very soft structure can cause bending at a significant level. Since the bending degrades the PDMS micropillar’s waveguiding capability, it can cause a decrease in the reflection level. The inset of Fig. 5a shows the optical micrograph of a multimode optical fiber section integrated with a HAR PDMS micropillar (height 412 µm, average diameter 18.4 µm, aspect ratio 22.4) at its end facet.

To test the operation of the soft optics-based contact sensor, we prepared a 40 µm-thick PDMS membrane and bonded it over a 2 mm-diameter PDMS chamber. Originally the tip of the PDMS micropillar was spatially separated from the PDMS membrane as shown in Fig. 5b, constituting the Non-Contact (NC) State. Then we pushed the PDMS membrane against the PDMS micropillar, gradually deforming the micropillar as shown in Figs 5c through 5e. Figure 5f shows the change in the reflection as a function of the level of deformation. Note that the measured reduction in the reflection level is approximately 15%, which is much less than the 75% change obtained in our previous work^10^. We attribute this decrease in the reflection level’s modulation depth to the low coupling efficiency between glass optical fiber and the flexible PDMS waveguide which degraded the reflection levels in the Contact and NC-states alike. The markings on Fig. 5f indicate the points at which Fig. 5b through 5e were captured. Overall, the microscope images indicate that the deformation starts to appear right below the microsphere at which the micropillar’s thickness becomes minimized. It then buckles to form a hook-like structure from which the reflection level diminishes rapidly. Once the buckling angle becomes 90° (Fig. 5d), the reflection level reaches its minimum and pinned. Further deformation and buckling, shown in Fig. 5e, did not cause additional reduction in the reflection level. Figure 5f also indicates that the contact between two soft structures, *i*.*e*., the PDMS micropillar and membrane, can generate clearly measurable differential signals in a reliable and repeatable fashion. Figure 5g shows the results of the contact sensing repeated over 5 cycles, which confirms that the contact sensing operation is fully reproducible and also the micropillar is robust enough to withstand multiple cycles. Since this contact sensing trial was mainly aimed at validating the utility of the new fabrication technique, further studies to rigorously relate the level of deformation and the change in the reflection level and quantification of the absolute force or pressure levels are left as future work.

## Discussion

The current work has greatly extended our previous scheme^10^ by enabling highly controllable drawing of PDMS micropillars on non-flat or highly space-limited surfaces. But at the same time, it may have given the impression that the fabrication throughput has become deteriorated because the new scheme draws the micropillars individually, on a pillar-by-pillar basis while the previous scheme was capable of drawing an array of micropillars simultaneously using a planar array of microspheres assembled on a planar surface. For fair comparison, two additional factors need to be considered. First, since the first scheme requires microspheres pre-assembled on a planar surface, the time and effort for the pre-process need to be factored into the throughput calculation as well. In microscale fabrication, the cost for such a pre-process can be significant. Second, it should be noted that the throughput of the previous scheme’s parallel drawing itself is not indefinitely scalable because the planar assembly must be maintained at perfect parallelism with the PDMS thin film below it in that scheme. Even a slight misalignment could lead to uneven interactions between the microspheres and the PDMS, resulting in failures or severe non-uniformities in the micropillar drawing. Achieving and maintaining parallelism are also difficult in microscale fabrication since they require specialized metrology schemes.

The comparison can be made on a process-by-process basis. As described in the Methods, major process steps of the new scheme include: (1) Preparation of a liquid-phase PDMS-filled pipette, (2) Position-controlled dispensing of PDMS microdroplets, (3) Vacuum-chucking of microspheres, (4) Position-controlled drawing of PDMS micropillars, (5) *In-situ* and post-curing of the micropillars, and (6) Releasing of microspheres. In terms of the time requirement, Step (5) claims the largest portion and it is common to both previous and current schemes. Step (1) does not take particularly longer time when compared with the time required for preparation of the PDMS thin film through spin-coating in the previous scheme. Step (6) in fact is almost instant in the new scheme, giving an advantage to it. Steps (2), (3), and (4) necessitate extensive use of micro-manipulators which may cost substantial amount of time. In practical realization, however, the processes can be automatized through the adoption of machine vision and computer control. It should be regarded as the price for the improved degree of freedom in micropillar drawing, rather than an unnecessary penalty.

With all these factors taken into consideration, we claim that the throughput levels of the two schemes are comparable and the new scheme ultimately prevails thanks to its extended degree of freedom in micropillar fabrication. We also believe that our microdroplet-based drawing technique can be applied to other types of materials and formation of final shapes more complicated than the cylindrical pillar. The key enabling factors will include finding the right material providing the proper levels of viscosity and curing rate in a balanced manner. In fact, the conventional, thin film-based drawing technique has already been utilized for materials other than PDMS^6,35^ and more advanced geometries such as a loop^36^.

## Conclusion

In this work, we demonstrated a new fabrication technique for high aspect-ratio PDMS micropillars. Existing techniques require totally flat, widely accessible substrate areas while allowing little *in situ* control over the micropillar’s structural characteristics. We overcame these limitations by directly drawing the micropillars from pipette-dispensed, isolated PDMS microdroplets using microspheres vacuum-chucked to pipettes as the drawing probes. By mounting the long and thin glass pipette on a 3-dimensional micromanipulator, we could access arbitrary positions on non-flat, highly space-constrained areas and produce PDMS micropillars at heights that can be controlled *in situ*. We verified the new technique’s utility by drawing PDMS micropillars on the end-facet of 125 μm-diameter optical fibers and also on the side of a 1 mm-diameter polymer fiber. The technique’s capability to control the micropillar’s height *in situ* can also be exploited to enable arrayed fabrication of highly dissimilar micropillars with narrow pillar-to-pillar spacing. As a validation, we also fabricated a row of three PDMS micropillars differing 70~100% in their heights but separated by only 900 μm. We also achieved fine tuning of the PDMS micropillar’s axial tapering through the adoption of UV-based PDMS curing. The new technique will be especially useful for hybridizing the PDMS micropillars with difficult-to-access and/or irregularly shaped structures to realize new sensing/actuation functionalities. As a proof-of-concept, we converted the optical fiber/micropillar hybrid described above into a soft optics-based contact sensor which will be useful for insertion and manipulation of intrabody biomedical devices.
